# Supplementary material for: Mechanisms governing the pioneering and redistribution capabilities of the non-classical pioneer PU.1
Source: Nat Commun. 2020 Jan 21;11:402. doi: 10.1038/s41467-019-13960-2 (PMC6972792; doi:10.1038/s41467-019-13960-2)
Supplement: Supplementary file 7 — Source data [file 41467_2019_13960_MOESM7_ESM.zip › Source_Data/FigureS2/FigureS2B_MotifScanOutput/KmeansCluster5/homerResults/motif9.similar.html]

motif9

## Information for motif9

A
C
G
T
T
G
A
C
A
C
G
T
G
A
T
C
C
T
G
A
G
A
C
T
C
T
A
G
C
G
T
A
A
C
T
G
C
T
G
A
C
G
T
A
G
T
A
C
G
C
A
T
A
T
C
G
  
Reverse Opposite:  

T
A
G
C
C
G
T
A
A
C
T
G
G
C
A
T
A
G
C
T
T
G
A
C
A
C
G
T
G
A
T
C
C
T
G
A
G
A
C
T
C
T
A
G
T
C
G
A
A
C
T
G
C
G
T
A
  

|  |  |
| --- | --- |
| p-value: | 1e-80 |
| log p-value: | -1.862e+02 |
| Information Content per bp: | 1.686 |
| Number of Target Sequences with motif | 197.0 |
| Percentage of Target Sequences with motif | 4.95% |
| Number of Background Sequences with motif | 297.1 |
| Percentage of Background Sequences with motif | 0.69% |
| Average Position of motif in Targets | 109.7 +/- 42.7bp |
| Average Position of motif in Background | 97.0 +/- 75.1bp |
| Strand Bias (log2 ratio + to - strand density) | 0.3 |
| Multiplicity (# of sites on avg that occur together) | 1.44 |
| Motif File: | file (matrix) reverse opposite |

### Similar de novo motifs found

|  |  |  |  |  |  |  |  |
| --- | --- | --- | --- | --- | --- | --- | --- |
| Rank | Match Score | Redundant Motif | P-value | log P-value | % of Targets | % of Background | Motif file |
| 1 | 0.972 | A G C T G T A C A G C T A G T C T C G A G A C T C T A G T C G A A C T G T C G A C G T A G A T C G C A T | 1e-76 | -175.969088 | 4.67% | 0.66% | motif file (matrix) |
| 2 | 0.949 | A C G T T G A C A C G T A G T C C T G A A G C T C T A G C G T A A C T G C G T A C G T A A G T C | 1e-70 | -161.202758 | 5.05% | 0.89% | motif file (matrix) |
| 3 | 0.880 | T G A C C G A T A G T C C T G A G A C T T C A G C G T A A C T G G C T A C G T A G T A C | 1e-60 | -139.608370 | 4.27% | 0.73% | motif file (matrix) |
| 4 | 0.872 | A C T G C G A T A C G T A G T C A C G T A G T C C T G A A G C T C T A G C G T A | 1e-57 | -131.859004 | 4.20% | 0.75% | motif file (matrix) |
| 5 | 0.781 | A C T G A G C T A G C T A T G C A G C T G A T C C T A G A G C T A C T G | 1e-29 | -68.400763 | 3.27% | 0.89% | motif file (matrix) |
| 6 | 0.780 | A C G T G T A C A C G T A G T C C T G A A G C T A C T G C G T A | 1e-29 | -68.079050 | 5.03% | 1.88% | motif file (matrix) |
